# *Pseudomonas aeruginosa* Coharboring *Bla*_KPC-2_ and *Bla*_VIM-2_ Carbapenemase Genes

**DOI:** 10.3390/antibiotics8030098

**Published:** 2019-07-20

**Authors:** Tatiana Pacheco, Rosa Helena Bustos-Cruz, Deisy Abril, Sara Arias, Lina Uribe, Jenny Rincón, Julio-Cesar García, Javier Escobar-Perez

**Affiliations:** 1 Grupo Evidencia Terapéutica, Clinical Pharmacology, Universidad de la Sabana, Chía 140013, Colombia; 2 Laboratorio de Genética Molecular Bacteriana, Universidad El Bosque, Bogotá 111321, Colombia

**Keywords:** *Pseudomonas aeruginosa*, carbapenems, carbapenemases, Verona Integron-encoded metallo-β-lactamase (VIM), *Klebsiella pneumoniae* carbepenemase (KPC), drug resistance

## Abstract

*Pseudomonas aeruginosa*, a bacterium commonly isolated from hospital settings, exhibits intrinsic resistance to a number of antibiotics and can acquire resistance during antibiotic therapy. Resistance towards carbapenems is increasing due to its overuse in the treatment of infections caused by extended-spectrum β-lactamase (ESBL) producing organisms. Nonetheless, carbapenems are essential for the treatment of high-risk infections and are one of the remaining weapons in the fight against “extreme drug resistance” of Gram-negative/positive bacilli. Herein, we describe a case report of infections caused by *P. aeruginosa* strains that carry *bla*_VIM-2_ and *bla*_KPC-2_ carbapenemase genes simultaneously, identified in five patients who were admitted to a high complexity health institution in Colombia. Molecular characterization included PCR screening for *bla*_KPC_, *bla*_GES_, *bla*_OXA-48_, *bla*_IMP_, *bla*_NDM,_ and *bla*_VIM_ carbapenemase and other resistance genes as well as analysis of the genetic relationships by genome macro-restriction and Pulsed-Field Gel Electrophoresis (PFGE) separation. In conclusion, these infections represent a major challenge to public health due to the risk of the infection spreading compounded by the fact that limited treatment options are available, thereby increasing the risk of increased morbidity and mortality.

## 1. Introduction

*Pseudomonas aeruginosa*, a Gram-negative, non-fermenting, rod-shaped bacterium, has become a significant concern in hospital-acquired infections as it infects immunocompromised patients. Incidences of *Pseudomonas aeruginosa* infections are on the rise worldwide due to its mechanisms of survival, adaptation, and resistance to different types of antimicrobials [1]. The National Healthcare Safety Network (NHSN) in the United States reported that from 2011 to 2014, *P. aeruginosa* was the sixth most common cause of hospital-acquired infections at 7.3% of all cases. The NHSN also reported that *P. aeruginosa* was the second most common cause of ventilator-associated pneumonia (VAP) (16.5%), as well as the most common multidrug-resistant (MDR) Gram-negative pathogen causing VAP. Furthermore, *P. aeruginosa* was also implicated in 10.3% of all catheter-associated urinary tract infections and 5.7% of all surgical site infections [2,3]. *P. aeruginosa* is a common pathogen worldwide and is one of the five most commonly isolated bacteria in hospitals in Colombia and other regions of Latin America [4,5]. The fact that *P. aeruginosa* is both intrinsically resistant and can acquire resistance to a number of antibiotics during therapy limits the available therapeutic options. Therefore, knowledge of the local resistance patterns is essential in order to establish the appropriate treatment strategies [3,4].

*P. aeruginosa* has multiple antibiotic resistance mechanisms that have been described as intrinsic, acquired, and adaptive [6]. Acquired resistance can occur as a result of mutation(s) or acquisition of exogenous resistance determinants and can be mediated by a number of mechanisms, including enzyme degradation, reduced permeability, and active efflux [7]. Intrinsic resistance is conferred by inherent structural or functional characteristics such as low outer membrane permeability, efflux of antimicrobials, and the production of antibiotic-inactivating enzymes [6]. Adaptive resistance, on the other hand, affects the lungs of patients via the formation of biofilms that serve as a barrier against antimicrobial infiltration [8].

Lasmid-mediated extended spectrum β-lactamases (ESBLs) have been implicated in acquired resistance owing to enzyme degradation, the most commonly described antimicrobial resistance mechanism. Temoneira (TEM), Sulfhydryl reagent variable (SHV), and cefotaximase (CTX-M) ESBLs have been reported in *P. aeruginosa*. Vietnam extended-spectrum β-lactamase (VEB) ESBLs are prevalent in *P. aeruginosa* strains in East Asia and are now also found in other regions. Pseudomonas extended resistant (PER) ESBLs, widely found in Turkey, confers a high-level of resistance on antipseudomonal cephalosporins [9,10]. Carbapenem resistance in *P. aeruginosa* can be a result of mutations, resulting in the loss of the OprD porin, but may also be a result of the production of carbapenemases such as Guiana Extended spectrum (GES), Imipenem metallo-β-lactamase (IMP), Verona Integron-encoded metallo-β-lactamase (VIM), Sao Paulo metallo-β-lactamase (SPM), and more recently, the *K. pneumoniae* carbapenemase (KPC) and New Delhi metallo-β-lactamase (NDM) [9,11].

KPC, a class A carbapenemase, was initially isolated from *K. pneumoniae* and has also been detected in most Enterobacteria [12]. However, in 2007, a *P. aeruginosa* isolate harboring the *bla*_KPC-2_ gene was identified in Colombia [13], and there have since been additional reports of such isolates in other countries [14,15,16,17,18]. The *bla*_KPC_ gene is mobilized on the 10 kb active Tn*3-*family Tn*4401* transposon, which is delimited by two 39-bp inverted repeat sequences [19]. The co-presence of *bla*_VIM-2_ and *bla*_KPC-2_ genes has been more frequently reported in the species of *K. pneumonia* [20] compared to *P. aeruginosa*, of which only three reports are available. The first report of the co-expression of *bla*_VIM-2_ and *bla*_KPC-2_ in *P. aeruginosa* occurred in Colombia in 2012 [21,22], followed by Chile [23], and later in Puerto Rico. It must be noted that in the latter, the *P. aeruginosa* isolate harbored KPC and IMP-8, simultaneously [24].

Here we report, for the first time, a case series of *P. aeruginosa* harboring VIM and KPC concurrently, producing two carbapenemases that represent a major public health challenge due to the risk of their successful dissemination and the limited classes of antibiotics that can be used for the treatment of these multi-drug resistant (MDR) isolates.

## 2. Results

### 2.1. Case 1

A 66-year-old patient was diagnosed with abdominal sepsis secondary to mesenteric ischemia. The patient was treated with piperacillin/tazobactam, meropenem, and fluconazole and required ventilatory support as well as a vasopressor. Due to poor clinical evolution, the patient required peritoneal lavage, which resulted in the isolation of MDR *P. aeruginosa* in the peritoneal fluid. Colistimethate (2,700,000 UI IV every eight h) was prescribed with follow-up of renal function. After four days of antibiotic therapy, the patient presented with clinical deterioration and cardiorespiratory arrest.

### 2.2. Case 2

The patient was 56 years old with a polytrauma Injury Severity Score (ISS) of 24 secondaries due to a high-energy traffic accident as an automobile driver, who suffered a complete sub-trochanter fracture of the left femur. After an initial clinical deterioration, the patient required an external tutor. During this surgery, in order to test for bone necrosis, the surgeon sent a bone sample for laboratory testing which was found to contain MDR *P. aeruginosa*. Consequently, broad-spectrum management with colistimethate (2,000,000 UI IV every eight h), rifampicin (600 mg once daily), and doripenem (1 g every eight h) was initiated. The patient completed antibiotic treatment and in-home hospitalization for 42 days.

### 2.3. Case 3

The patient was 84 years old with a history of Wegener′s disease, a right hip replacement, and infection at the operative site by *Proteus mirabilis* that was treated with meropenem for 21 days. A surgical lavage was performed due to the patient’s clinical decline resulting in the identification of carbapenem-resistant *P. aeruginosa* in bone and blood cultures. Antibiotic treatment was started with colistimethate (2,000,000 UI IV every eight h), doripenem (1 g every eight h), and rifampicin (600 mg every 12 h) for 42 days, and the hip implant was also removed. On day 10 of the treatment, the patient showed clinical deterioration and later died.

### 2.4. Case 4

The patient was 57 years old with a history of benign prostatic hyperplasia necessitating a long-term urinary catheter, with complicated diverticular disease that required subtotal colectomy. During hospitalization, the patient required multiple invasive medical devices and approximately seven surgical lavages. The patient presented bacteraemia caused by *P. aeruginosa* and received treatment with colistimethate (2,400,000 UI IV every eight h) and doripenem (1 g every eight h) for 10 days with favorable clinical evolution and negative control cultures. The patient was discharged after two weeks of hospitalization.

### 2.5. Case 5

A 29-year-old patient with a history of epilepsy and Down syndrome presented with pulmonary septic shock due to carbapenemase-producing *P. aeruginosa* that was treated with colistimethate and doripenem. Following clinical improvement, the patient was discharged after 10 days but then was readmitted at day 15 with a systemic inflammatory response and deterioration of the respiratory pattern. Cultures showed a urine culture with MDR *P. aeruginosa*. Colistimethate (1,500,000 UI IV every eight h), doripenem (1 g every eight h), and Fosfomycin were prescribed for 12 days. The patient presented with clinical deterioration and died during hospitalization. The most important characteristics of the five patients are shown in Table 1.

All five isolates were resistant to meropenem, imipenem, gentamicin, ciprofloxacin, trimethoprim/sulfamethoxazole, and piperacillin/tazobactam but remained susceptible to colistin (0.5 µg/mL). Analysis of the meropenem and doripenem minimal inhibitory concentration (MIC) showed that all isolates reached a MIC value of 1024 µg/mL and 512 µg/mL, respectively. Molecular characterization revealed that the five isolates simultaneously harbored the *bla*_VIM-2_ and *bla*_KPC-2_ carbapenemase genes, and the *bla*_TEM_ and *aac(6’)-lb* genes were also detected. In *P. aeruginosa,* the *bla*_KPC-2_ gene is mainly found on both complete and truncated Tn*4401b* transposons, within two different plasmid backbones (IncU and IncP-6 incompatibility groups). We recently reported a *P. aeruginosa* isolate (24Pae112) that contained a double chromosomal insertion of the Tn*4401b*-*bla*_KPC-2_ transposon, which was inserted into the new pathogenicity island (PAGI-17). We designed primers to amplify specific DNA fragments of these three genetic platforms (see Materials and Methods); however, they were not identified in the *P. aeruginosa* isolates, suggesting that the *bla*_KPC-2_ gene could be mobilized in a different platform.

**Figure 1:**
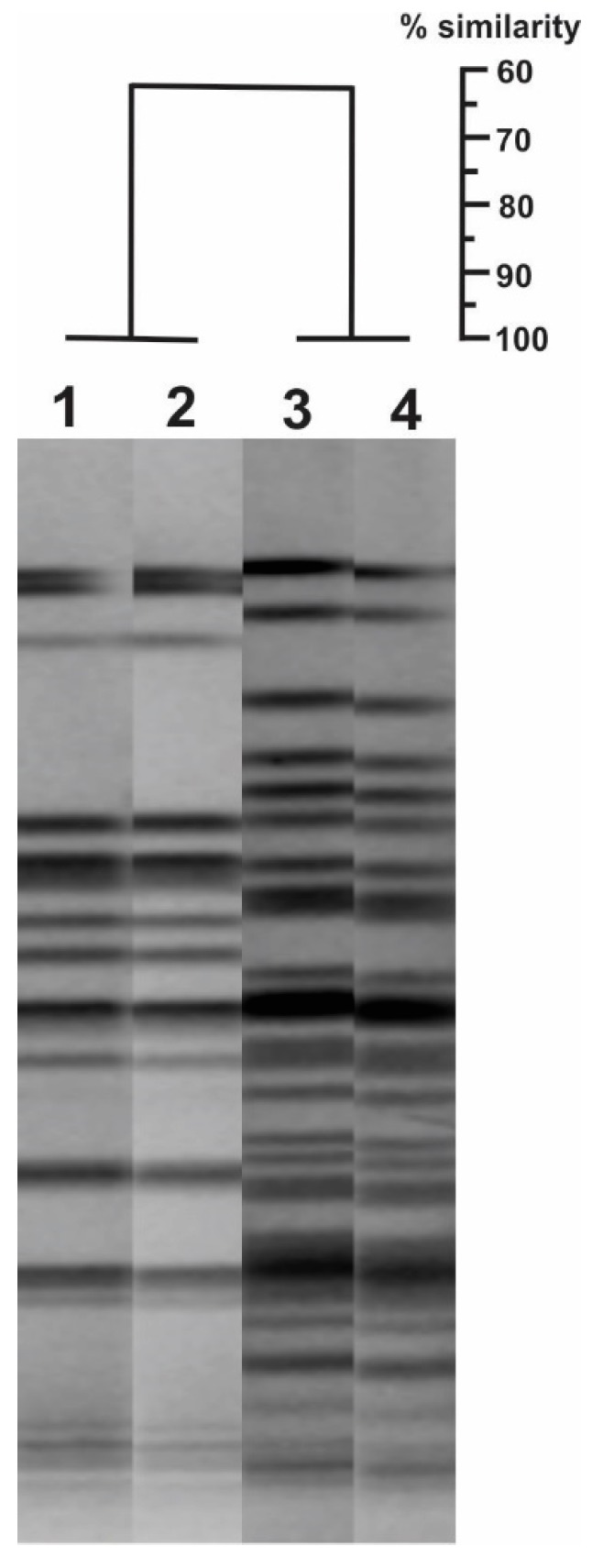
Genetic relationship by Pulsed-Field Gel Electrophoresis (PFGE) of the *Pseudomonas aeruginosa* isolates that harbor the double chromosomal insertion of the *bla*_KPC-2_ transposon (lanes 1 and 2) and those carrying the *bla*_VIM-2_ and *bla*_KPC-2_ genes simultaneously (lanes 3 and 4). GelCompar II program (Applied Maths NV) was used, with a tolerance position of 1.5% and a Dice coefficient of 1.0%.

The Pulsed-Field Gel Electrophoresis analysis revealed that the five isolates had an identical pulsotype, but they were different from the 24Pae112 isolate pulsotype (Figure 1), suggesting that this carbapenemase was acquired through unrelated clones. The *intl1* gene and the presence of *bla*_VIM_ gene into the class I integron was confirmed by PCR.

## 3. Discussion

Herein, we present a series of five intensive care unit (ICU) hospitalized patients with MDR *P. aeruginosa* infections with a very high MIC to carbapenems and harboring the *bla*_KPC-2_ and *bla*_VIM-2_ genes at the same time. Patients ranged in age from 29 to 84 years, and the majority were men with diverse comorbidities as well as different sites of infection. Only two patients shared a bone infection, and all treatments followed the institutional recommendation for each case. All patients received an antibiotic combination of two or three drugs with all five regimens including colistimethate. The mortality was high (60%). Bacterial strains producing two carbapenemases represent a major public health challenge due to the risk of their successful spread and difficulty of treating the infections caused by these MDR isolates [25]. To the best of our knowledge, there are no case series reports regarding this significant issue in *P. aeruginosa*.

Co-harboring of carbapenemases is a genetic event that, in recent years, has increased in its frequency due to the increased clinical usage of carbapenems. The co-expression of VIM and KPC enzymes has been reported more frequently in species of *K. pneumoniae* [22,26,27]. However, the first *P. aeruginosa* isolate co-harboring VIM-2 and KPC-2 was reported in Colombia in 2012 and later in Chile [23]. *P. aeruginosa* isolates harboring KPC and IMP-8 simultaneously were found in Puerto Rico [24]. In Colombia, the circulation of the *bla*_KPC-2_*-*containing *P. aeruginosa* isolates initiated a national public health problem because such isolates have increased in their frequency since 2007 [23]. In 2012, an analysis of 43 carbapenemase-producing *P. aeruginosa* isolates recovered from seven Colombian cities showed that there was a higher frequency of isolates with *bla*_VIM-2_ with respect to those with *bla*_KPC-2_ (33 vs. 9) [28]. In 2014 and 2015, two studies found a similar frequency of both the *bla*_KPC-2_*-* and *bla*_VIM-2_*-*containing *P. aeruginosa* isolates [20,24]. A recent study conducted in seven healthcare institutions in Bogota, Colombia, found that the *bla*_KPC-2_*-*containing *P. aeruginosa* isolates were the most frequent (4:1 ratio between *bla*_KPC-2_ and *bla*_VIM-2_, respectively) (data in publication process). Currently, the spread of *P. aeruginosa* co-expressing KPC and VIM presents a significant public health challenge.

The global spread of carbapenem resistance among Gram-negative organisms is explained by horizontal gene transfer, although the first carbapenemases described were chromosomally encoded and species-specific [29]. Latin America is not immune to this dissemination. Limited resources for performing the appropriate microbiological assays in the vast majority of clinical laboratories lead to an underestimation of the real problem [22]. A recent review of the epidemiology of carbapenemases in Latin America and the Caribbean identified an increased frequency of reports in both regions. This clearly illustrates the ability of these enzymes to successfully spread, becoming endemic in some countries [30].

Therapeutic options for patients with MDR bacterial infections are scarce. Treatment options include carbapenems with lower MIC and adequate penetrance to the site of infection, tigecycline, fosfomycin, amikacin, polymyxins, and some authors recommend rifampicin and daptomycin [31]. Recent advances include ceftazidime/avibactam, which was not available for use in Colombia when isolates for this study were collected. In this regard, the use of the latter is limited by the presence of metallo-β-lactamases [32], as in our cases.

These findings underscore the importance of conducting campaigns for preventing the spread of these types of carbapenemase-producing pathogens not just in our institution, but in healthcare facilities in Colombia and Latin America, especially because of their rapid dissemination.

## 4. Materials and Methods

### 4.1. Bacterial Isolates and Susceptibility Profile

The *P. aeruginosa* isolates were recovered from different samples using standard microbiological techniques [33] and were stored in Brain Heart Infusion (BHI) broth (Oxoid-Thermo Scientific^®^, Hampshire, United Kingdom) supplemented with 15% glycerol at −80 °C until use. Bacterial identification and the susceptibility profiles to meropenem, imipenem, ceftazidime, gentamicin, amikacin, ciprofloxacin, trimethoprim/sulfamethoxazole, and piperacillin/tazobactam were determined by automated VITEK^®^2 systems using the breakpoints defined by the Clinical and Laboratory Standards Institute, 2018 [34]. The *P. aeruginosa* (ATCC^®^ 27853^TM^) strain was used as a susceptibility control (American Type Culture Collection, https://www.atcc.org/products/all/27853.aspx). The MIC to meropenem and colistin was established by the broth dilution method.

### 4.2. Detection of Resistance Genes

The *bla*_TEM_*, bla*_SHV,_ *bla*_CTX-M,_ *bla*_FOX_, *bla*_ACT_, *bla*_MIR_ *bla*_ACC_, *bla*_DHA_, *bla*_CMY_, and *bla*_MOX_ genes were assessed using two multiplex PCR described previously [35]. The *bla*_IMP_*, bla*_OXA-48_*, bla*_VIM_*, bla*_GES_*, bla*_KPC_*, bla*_NDM_ carbapenemase genes were assessed by multiplex PCR in accordance with previously reported conditions [36]. The OXA-derived carbapenemase genes (*bla*_OXA-23_, *bla*_OXA-24_, *bla*_OXA-43_, *bla*_OXA-51*,*_ and *bla*_OXA-58_) were also assessed by multiple PCR [37,38]. In addition, a screening of genes related to aminoglycoside and fluoroquinolone resistance was performed (*aac(6´)-lb, aac(6´)-lb-cr, qnrA, qnrB*, *qnrS,* and *mcbG*) [39]. The *bla*_KPC-2_ and *bla*_VIM-2_ variants were determined by sequencing of complete genes using the dideoxy chain termination method [40]. Finally, the *intl1, intl2*, and *intl3* genes were assessed using primers previously reported [41] (Gene ID: 13906549 for *intl1* gene, and GenBank accession number: KJ184348.1 and BBA94100.1 for *intl2* and *intl3* genes, respectively).

### 4.3. Establishment of the Genetic Relationship by Pulsed-Field Gel Electrophoresis (PFGE)

The genetic correlation between the isolates was determined by genome macro-restriction using the *Spe*I enzyme (Promega, Madison WI, USA) and PFGE separation according to previously reported methods [42]. Briefly, one colony of each isolate was grown in 5 mL of BHI broth at 37 °C for 12 h (2 × 10^8^ cells). The bacteria were harvested by centrifugation, washed twice, and resuspended in 2 mL of TE buffer (0.2 M Tris-HCl, 20 mM EDTA (pH 7.5). Then 200 µL of bacterial suspension were mixed with 200 µL of a 1.5% agarose solution and deposited into a casting mold. The embedded cells in the agarose inserts were subjected to detergent and enzymatic lysis (50 mM Tris, 50 mM EDTA-pH 8.0, 1% Sarcosyl, and 400 µg proteinase K). The insert was washed and stored in TE buffer at 4 °C until it was ready to use. The DNA-containing agarose slices (2 mm) were subjected to digestion using 12 U *Spe*I enzyme (Promega) in buffer Tango^®^ at 37 °C for five h. The DNA-fragments generated were separated using a CHEFII Pulsed Field Electrophoresis system (Biorad, California CA, USA) for 23 h at 14 °C, 120 V (6 V/cm), and with an initial and final time of 6.8 s and 35.4 s, respectively.

### 4.4. Assessment of the Genetic Platforms Mobilizing the Bla_kpc_ Gene

Currently, three genetic platforms have been reported mobilizing the *bla*_KPC-2-_positive Tn*4401b* transposon in *P. aeruginosa,* two different plasmid backbones (8 kb and 31.5 kb) belonging to the IncU and IncP-6 incompatibility groups [43,44], and a double chromosomal insertion within a new genomic island named PAGI-17 [45]. The specific primers were designed to amplify DNA fragments for each platform (Table 2). The optimal conditions for each PCR were established.

### 4.5. Ethical Approvals

Isolates were obtained as part of routine diagnostic testing and analyzed anonymously. The study was approved by the Research Ethics Committee of the Faculty of Medicine at Universidad de La Sabana (Acta 410, 9 June 2017).

## 5. Conclusions

We report for the first time a case series of infections caused by *P. aeruginosa* that concurrently harbor VIM and KPC. This represents a major public health problem considering the risk of an outbreak of such an infection in combination with the fact that we have limited therapeutic tools to treat such infections could be calamitous.

## Figures and Tables

**Table 1:** Relevant clinical characteristics of patients.

Patient	Date of Isolation	Age (Years)	Gender	Comorbidities	Site of Infection	Treatment	Death (Yes/No)	MIC * (µg/mL)							
								MEM	DOR	CAZ	TZP	GEN	CIP	SXT	CST
1	8 April 2017	66	Male	None	Abdomen	Colistimethate + Doripenem	Yes	1024	512	>256	>512	>256	32	>256	0.5
2	25 March 2017	56	Male	Polytrauma	Bone	Colistimethate + Doripenem + Rifampin	No	1024	512	>256	>512	>256	16	>256	0.5
3	18 April 2017	84	Female	Total hip replacement	Bone	Colistimethate + Doripenem + Rifampin	Yes	1024	512	>256	>512	>256	32	>256	0.5
4	22 April 2017	57	Male	Benign prostatic hyperplasia	Blood	Colistimethate + Doripenem	No	1024	512	>256	>512	>256	32	>256	0.5
5	12 July 2017	29	Male	Epilepsy, Down Syndrome	Urine	Colistimethate + Doripenem + Fosfomycin	Yes	1024	512	>256	>512	>256	32	>256	0.5
**MIC of ATCC27853**							-	0.25	0.12	4	4	1	0.12	16	0.5

* MEM, meropenem; DOR, doripenem; CAZ, ceftazidime; TZP, piperacillin-tazobactam; GEN, gentamicin; CIP, ciprofloxacin; SXT, trimethoprim/sulfamethoxazole; and CST, colistin.

**Table 2:** List of primers used in this study.

Code	DNA Sequence	Amplicon Size (bp)	Specific Target	Accession Number (GenBank)
GN634	AAACGTGAACCTGGCTTTGT	183	*Orf6*-IncP-6	KC609323.1
GN635	CGCATCCACAAATGACAATC		*Orf6*-IncP-6	
GN636	TCCGCCTTTTGCTTCTCGAT	545	*repA*-IncU	KC609322.1
GN637	GAGCAGATGCCAACAGTCCT		*repA*-IncU	
GN626	GCAGCAAGAACTGGGACGA	835	*arsC3*	NZ_CP029605
GN656	TTTGGTGCGTGTTGCGAAG		*tnpR*	
GN628	GATGAAACGGCTGATTGCCC	953	*tnpA*	
GN657	TACAGGCCGACCGATACCA		*acr3*	
GN630	TACAGCGTGTCGTACTGCTT	960 *	*parB*-like gene	
GN658	ACCTACTTTGAGGCCGATGAG	994 **	*acrA*	

* Expected size when used in combination with GN628. ** Expected size when used in combination with GN656.
